# Asymptomatic abdominal aortic stenosis detected by unenhanced computed tomography before angiography in acute myocardial infarction

**DOI:** 10.1002/ccr3.1780

**Published:** 2018-08-29

**Authors:** Toshihiro Suga, Takashi Hatori, Yuko Suga, Mikoto Yoshida, Keita Oyama

**Affiliations:** ^1^ Department of Cardiology Gunma Chuo Hospital Maebashi city Gunma Japan

**Keywords:** asymptomatic abdominal aortic stenosis, myocardial infarction, unenhanced computed tomography

## Abstract

Although rapid treatment is important, unenhanced computed tomography before angiography is quick and can detect myocardial infarction induced by aortic dissection and also asymptomatic abdominal aortic stenosis in acute myocardial infarction cases.

The usefulness of unenhanced computed tomography (UCT), which has been reported to be useful for detecting aortic dissection (AD)^1^ before angiography in acute myocardial infarction (AMI) cases, remains unclear. A 52‐year‐old diabetic woman without a history of intermittent claudication was admitted following chest pain. AMI was diagnosed from electrocardiography and echocardiography. Since the femoral artery pulse was weak, UCT was performed to exclude AD‐induced AMI. Severe abdominal aortic calcification suggesting stenosis was detected by UCT coincidentally (Figure 1A). Emergency angiography via the radial artery revealed total occlusion of the left circumflex artery and abdominal aortic stenosis (AAS) with a collateral vessel (Figure 1B). An intra‐aortic balloon pumping (IABP) system via the femoral artery could pass the AAS, and IABP support resulted in stable hemodynamics and percutaneous coronary intervention was performed successfully.

**Figure 1:**
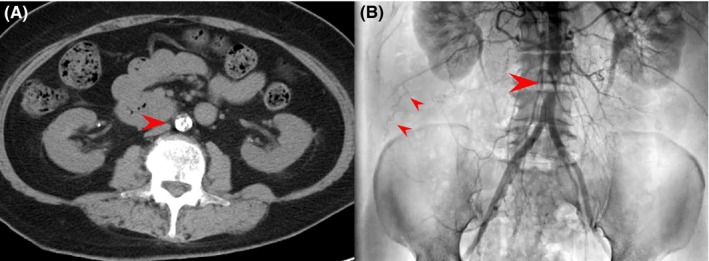
A, Abdominal aortic calcification suggesting stenosis. B, Abdominal aortic stenosis (big arrow) with collateral vessel (small arrow)

This case showed the usefulness of UCT before angiography in detecting asymptomatic AAS accompanied by AMI, which has been rarely reported. AAS existence could inhibit IABP and influence the treatment strategy. Diabetes or collateral vessels may cause asymptomatic AAS.^2^ Although rapid treatment is important, UCT before angiography, which is not time‐consuming, and can detect not only AD‐induced AMI but also AAS, may be useful in AMI cases.

## CONFLICT OF INTEREST

None declared.

## AUTHOR'S CONTRIBUTIONS

TS: collected data, drafted, and revised the manuscript. TH, YS, MY, and KO: helped TS collect data, provided intellectual input, and revised the manuscript.

## References

[1] Kurabayashi M , Okishige K , Ueshima D , et al. Diagnostic utility of unenhanced computed tomography for acute aortic syndrome. Circ J. 2014;78:1928‐1934. 24909890 10.1253/circj.cj-14-0198

[2] Alessandro M , Marco B , Paola C , et al. When collateral vessels matter: asymptomatic Leriche syndrome. Clin Case Rep. 2015;11:960‐961. 10.1002/ccr3.390 PMC4641484 26576282

